# Clinicopathologic Characteristics of Epstein–Barr Virus-Associated Gastric Cancer Over the Past Decade in Japan

**DOI:** 10.3390/microorganisms7090305

**Published:** 2019-09-01

**Authors:** Ayaka Yanagi, Jun Nishikawa, Kanami Shimokuri, Takuya Shuto, Tatsuya Takagi, Fumiya Takagi, Yuki Kobayashi, Misa Yamamoto, Osamu Miura, Hideo Yanai, Yutaka Suehiro, Takahiro Yamasaki, Hironori Yoshiyama, Isao Sakaida

**Affiliations:** 1 Faculty of Laboratory Science, Yamaguchi University Graduate School of Medicine, 1-1-1 Minami-Kogushi, Ube, Yamaguchi 755-8505, Japan; 2 Hofu Institute of Gastroenterology, Hofu 747-0801, Japan; 3 Department of Clinical Research, National Hospital Organization Kanmon Medical Center, 1-1 Sotoura, Chofu, Shimonoseki, Yamaguchi 752-8510, Japan; 4 Department of Oncology and Laboratory Medicine, Yamaguchi University Graduate School of Medicine, 1-1-1 Minami-Kogushi, Ube, Yamaguchi 755-8505, Japan; 5 Department of Microbiology, Shimane University School of Medicine, 89-1 Enyacho, Izumo, Shimane 693-8501, Japan; 6 Department of Gastroenterology and Hepatology, Yamaguchi University Graduate School of Medicine, 1-1-1 Minami-Kogushi, Ube, Yamaguchi 755-8505, Japan

**Keywords:** Epstein–Barr virus (EBV), gastric carcinoma, carcinoma with lymphoid stroma, lymph node metastasis, immune checkpoint inhibitor

## Abstract

Epstein–Barr virus (EBV) is a ubiquitous human herpes virus, but related with several types of malignancies. Among EBV-related malignancies, EBV-associated gastric carcinoma (EBVaGC) has the largest patient’s number. We screened for EBV infection in 1067 GC lesions of 1132 patients who underwent surgical resection from 2007 to 2017 in Japan and examined clinicopathological features of EBVaGC. EBV infection was detected by *in situ* hybridization with EBV-encoded small RNA 1(EBER-1 ISH). EBV was infected in 80 GC lesions (7.1%). Mean age was significantly lower in patients with EBVaGC than with EBV-negative GC. EBVaGC was more frequent in men than in women. EBVaGC was found twice as frequent in the upper or middle stomach as in the lower stomach. Early EBVaGC was more frequent, and submucosally invaded cases were dominant. The presence of lymphatic vessel invasion was less in EBVaGC, but frequency of lymph node metastasis was similar. Carcinoma with lymphoid stroma (CLS) was found in 3.8% (43/1132) of all lesions with 60.5% of EBV positivity. The synchronous or metachronous multiple GC was frequent in EBVaGC. We clarified clinicopathologic characteristics of EBVaGC over the past decade in Japan. EBV infection should be examined in gastric cancer cases showing these characteristics.

## 1. Introduction

Epstein–Barr virus (EBV) is a ubiquitous human herpes virus discovered from a Burkitt lymphoma cell. EBV is associated with a variety of tumors derived from B cells, such as Burkitt lymphoma, post-transplant lymphoproliferative disease, and Hodgkin’s disease. EBV is also detected from epithelial tumors, including nasopharyngeal carcinoma and gastric carcinoma (GC). Although not frequent, the virus is sometimes associated with peripheral T-cell lymphomas and nasal natural killer T-cell lymphomas [1,2].

Gastric cancer is the fifth frequently diagnosed cancer and the third leading cause of cancer deaths worldwide [3]. EBV-associated GC (EBVaGC) is about 10% of all GC worldwide [4,5]. EBVaGC is not endemic like EBV-related Burkitt lymphoma and nasopharyngeal carcinoma. Among EBV-related malignancies, EBVaGC is the most common. However, EBVaGC has not received clinical attention because there is no specific treatment for this malignancy.

The Cancer Genome Atlas (TCGA) 2014 divided gastric cancer into four types according to characterization based on molecular biology: EBVaGC, microsatellite unstable tumors, genomically stable tumors, and chromosomally unstable tumors. EBVaGC characteristically shows recurrent PIK3CA mutations, extreme DNA hypermethylation, and overexpression of each of JAK2, PD-L1, and PD-L2 [6]. Therefore, demethylating agents, PI3K or JAK2 inhibitors, and immune checkpoint inhibitors may be effective in the treatment of EBVaGC [7]. Selection of therapy based on the mechanism of tumor development is required, and it is important to diagnose EBVaGC prior to treatment.

EBVaGC shows that infrequent lymph node metastasis and EBV positivity in gastric cancers confers a better survival advantage than EBV-negative cancers [8,9]. Minimally invasive endoscopic treatment has been applied to early gastric cancer. Because endoscopic treatment is a local treatment inside the stomach, gastric cancer without lymph node metastasis is the principal indication [10]. The number of reports that mention infrequent lymph node metastasis in EBVaGC is increasing, especially in early-stage cancer [11,12]. Early-stage EBVaGC can be an indicator for more extensive endoscopic treatment than other types of gastric cancer.

Because important features of EBVaGC related to treatment strategies have been reported, it is necessary to diagnose EBVaGC before treatment. Thus, we examined EBV infection in a series of about 1000 gastric cancers over the past decades in Japan to clarify the clinicopathologic features of EBVaGC.

## 2. Materials and Methods

### 2.1. Patients

The subjects were 1067 GC lesions of 1132 patients with gastric cancer who underwent surgical resection at Yamaguchi University Hospital and Hofu Gastroenterology Center from 2007 to 2017. This retrospective study was approved by the institutional review board of Yamaguchi University Hospital (approval number: H26-119-3) (Date: 24 December 2014).

### 2.2. EBV-Encoded Small RNA 1 (EBER-1) in Situ Hybridization

The presence of EBV was determined using *in situ* hybridization (ISH) with EBV-encoded small RNA 1 (EBER-1), which is known to be present in large amounts in EBV-infected cells. EBER-1 was detected with a biotin- or digoxigenin-labeled 30-base oligomer using previously described procedures [13]. Paraffin-embedded 4-mm sections were deparaffinized, rehydrated, predigested with pronase, prehybridized, and then hybridized overnight at 37 °C. After washing with 0.5× SSC, hybridization was detected according to the manufacturer’s instructions. EBVaGC was diagnosed by EBER-1 ISH, where most of tumor cell nuclei were positively stained. 

### 2.3. Evaluation of Clinicopathologic Findings

The locations of the GCs were divided into three areas: upper, middle, and lower stomach. We had 28 GC lesions developed in the remnant stomach after partial gastrectomy. Histologic types were divided into differentiated and undifferentiated types. Carcinoma with lymphoid stroma (CLS), which is considered to be a pathological feature of EBVaGC, was classified separately. Lesions with cancerous invasion limited to the mucosa or submucosa were classified as early, and lesions with deeper invasion as advanced. The macroscopic types of the GCs were classified according to the Japanese Classification of Gastric Carcinoma [14].

### 2.4. Statistics

Data were analyzed using the Mann–Whitney U test. A *p* value of 0.05 was considered significant.

## 3. Results

**Figure 1:**
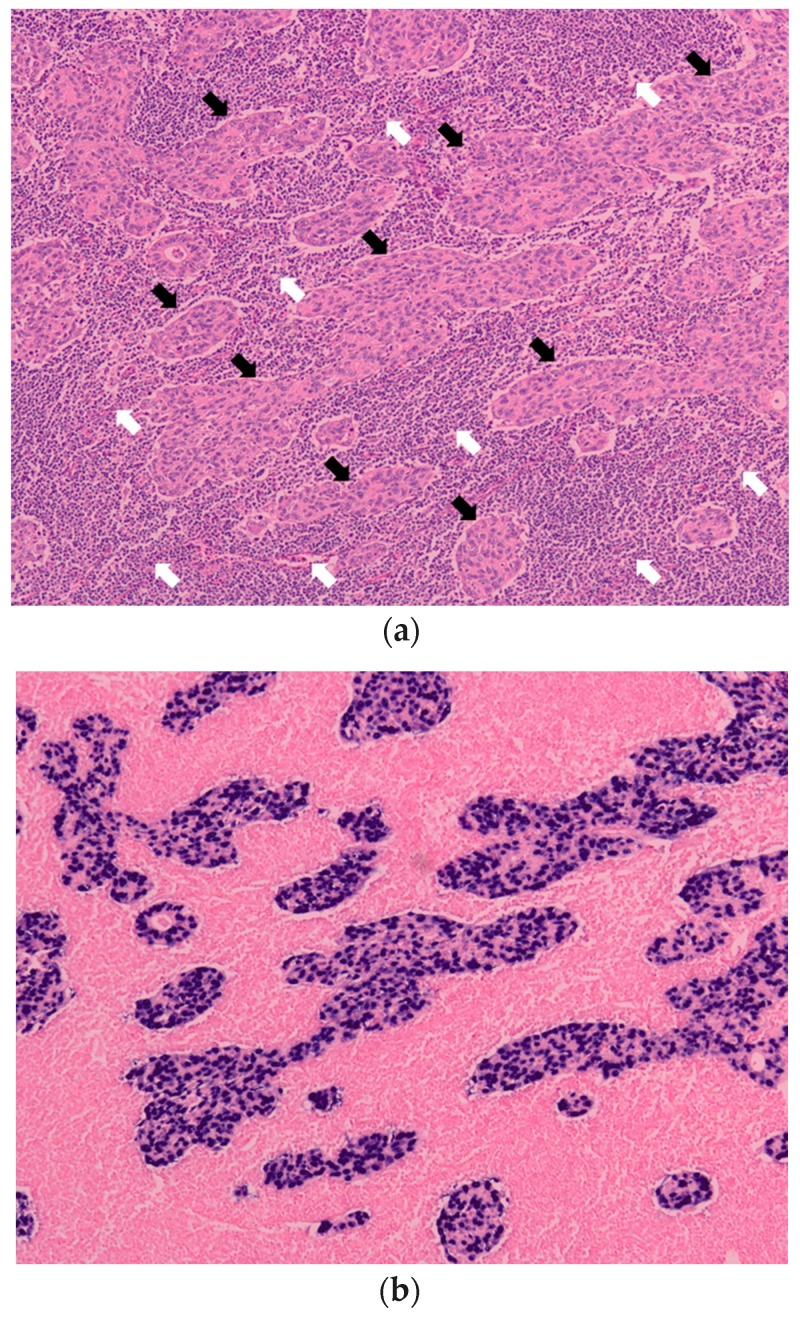
Histologic characteristics of Epstein-Barr virus-associated gastric carcinoma (EBVaGC). (**a**) Histology of carcinoma with lymphoid stroma (CLS). Undifferentiated adenocarcinomas (black arrows) are surrounded by infiltrating lymphocytes (white arrows). (H&E staining); (**b**) EBV-encoded small ribonucleic acid 1 in situ hybridization (EBER-1 ISH). Signals of EBER-1 are observed in nuclei of the carcinoma cells indicated by black arrows in (**a**). Infiltrating lymphocytes in the stroma indicated by white arrows in (**a**) are EBER-1 negative. Magnification (**a**) and (**b**)(×100).

**Figure 2:**
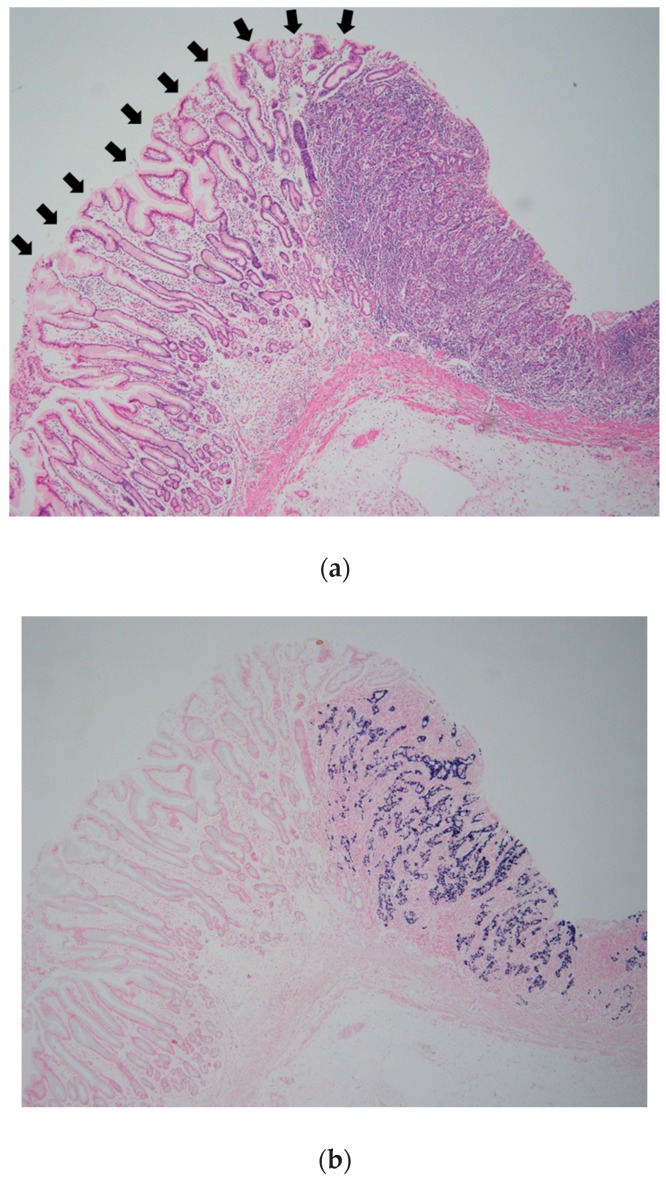
Epstein–Barr virus-associated gastric carcinoma (EBVaGC). (**a**) Moderately differentiated adenocarcinoma is composed of irregular tubules and moderate lymphocytic infiltration. Black arrows indicate non-cancerous epithelium; (**b**) Dark-blue signals of EBV encoded small RNA 1 (EBER-1) are observed in cancerous area. Epithelial cells in the non-cancerous mucosa indicated by black arrows in (**a**) are EBER-1 negative. Magnification (**a**) and (**b**) (×40).

A large series of 1132 GC lesions in 1067 consecutive patients was studied. EBV was detected in 80 GC lesions (7.1%) by EBER-1 ISH. EBER-1 signals were observed uniformly in the nuclei of tumor cells in these lesions. EBV was not present in infiltrating lymphocytes of the stroma. Epithelial cells in the adjacent mucosa were EBER-1 negative (Figure 1 and Figure 2).

The mean age of the patients with EBVaGC was significantly lower than that of patients with EBV-negative gastric cancers. EBVaGC was mostly detected in male patients, and it arose twice as frequent in the upper or middle stomach as in the lower stomach. The rate of early cancer was significantly high for EBVaGC, and submucosally invaded cancers were predominant. The degree of tumor differentiation and macroscopic type were similar. EBVaGC showed a significantly infrequent lymphatic vessel invasion and the frequency of lymph node metastasis was similar. The frequency of synchronous or metachronous multiple GC was higher in EBVaGC than in the EBV-negative cancers (Table 1).

**Figure 3:**
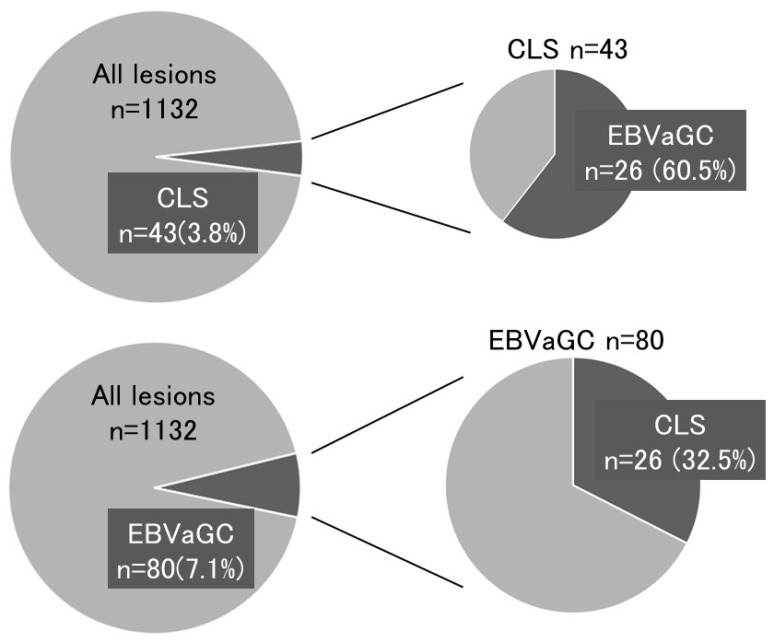
Relationship between Epstein–Barr virus-associated gastric carcinoma (EBVaGC) and carcinoma with lymphoid stroma (CLS).

Carcinoma with lymphoid stroma (CLS) was classified separately. CLS was found in 3.8% (43/1132) of all lesions, and 60.5% (26/43) of CLS was positive for EBV Figure 3 and Table 1.

## 4. Discussion

EBV was detected in 80 GC lesions (7.1%). The results shown in this study were almost identical to the previous meta-analytical examinations [5]. In our cohort, early stage EBVaGC was more frequent, and submucosally invaded cancers were dominant. Synchronous or metachronous multiple GC were frequently found. Because frequent recurrence of EBVaGC is an important issue, careful follow-up of the remnant stomach after surgical or endoscopic resection of EBVaGC is necessary [15,16]. 

The characteristic pathological feature of EBVaGC is lymphocyte infiltration (CLS) [17,18,19,20]. Therefore, if this histopathologic feature is observed in the clinical setting, the patient is examined for EBV infection. Patients are also examined for EBV infection when endoscopic and endosonographic features reflecting the histology of CLS are found. As EBVaGC is comprised of a poorly differentiated tumor mass that accompanies infiltrating lymphocytes in a submucosal or deeper layer, EBVaGC exhibits a submucosal tumor-like morphology on endoscopic observation or a well-defined hypoechoic mass in the submucosa on endosonographic examination [21,22]. Watanabe et al. showed that CLS was found in 4% of gastric carcinomas removed surgically [23]. The present cohort showed frequencies of 3.8% for CLS and 7.1% for EBVaGC (Figure 3), indicating that EBVaGC is made up of two histological lesions, CLS, and ordinary gastric cancer at a similar frequency. Thus, if EBVaGC is searched simply according to the characteristics of CLS, half of EBVaGC cases will be undetected.

EBVaGC is classified into one of the molecular subtypes in TCGA, and overexpression of PD-L1 has been shown in this cancer [24,25,26,27,28]. Responders to immune checkpoint inhibitors show high PD-L1 expression in tumor cells, high lymphocyte infiltration in tumor stroma, and high IFN-γ content in tumors [29,30,31,32]. EBVaGC exhibits all of these characteristics of responders. In a prospective phase II clinical trial of pembrolizumab, dramatic responses were observed in patients with EBVaGC and microsatellite instability-high (MSI-H) tumors [33]. It is desirable to check for EBV infection prior to chemotherapy because EBVaGC can become a good indication to use immune checkpoint inhibitors. Unresectable recurrent gastric cancer that will be treated by chemotherapy need to be investigated for EBV infection.

In the present study, the rate of early gastric cancer was high in EBVaGC, with 33 cases of submucosal EBVaGC. Endoscopic local treatment has recently been expanded to treat submucosally invaded gastric cancer and can be applied to patients without lymph node metastasis. The rate of lymph node metastasis of submucosal gastric cancer is usually 20%, whereas that of the current cases of EBV-positive submucosal gastric cancer was 12.1%. Regarding to the submucosal cancer without vascular invasion, only 1 case (3.0%) was found to show lymph node metastasis. According to Tokunaga et al., there was no lymph node metastasis in early EBVaGC [11]. Osumi et al. analyzed 847 consecutive patients with submucosal GC without vascular invasion in a multicenter observational study and showed that EBVaGC accounted for 11.3% (96/847) of cases, and lymph node metastasis was less frequent in EBVaGC versus non-EBVaGC (1 of 96, 1.0% vs. 71 of 751, 9.5%) [34]. For the treatment of submucosally invaded gastric cancer with EBV and without lymphovascular invasion, endoscopic local treatment might be applied instead of surgical resection.

Endoscopic examination is mainly used for diagnosing GC. However, treatment strategies are decided by pathologic diagnosis of biopsy samples. The macroscopic findings in the present study were too diverse to find specific features. It is not easy to predict EBVaGC based on clinical characteristics. Only half of EBVaGC can be diagnosed, if there were the pathological findings such as CLS. Because it is not practical to perform EBER-1 ISH for all GC patients, we should carefully focus on cases where treatment strategies may change depending on the EBV infection. Patients with unresectable recurrent gastric cancer and off-label lesions after endoscopic treatment should be examined for EBV infection. 

## 5. Conclusions

We clarified the clinicopathological features of EBVaGC in a series of about 1000 gastric cancers over the past decades in Japan. EBV infection should be examined for gastric cancer showing certain characteristics.

## Figures and Tables

**Table 1:** Clinicopathologic features of Epstein–Barr virus (EBV)-associated gastric cancer.

Feature	Total	EBV Positive	EBV Negative	*p* Value
Sex				
Male	735 (68.8%)	61 (88.4%)	674 (67.5%)	0.022
Female	332 (31.2%)	8 (11.6%)	324 (32.5%)	
Age (yrs, mean)		65.6	68.9	<0.001
Location				
U	228 (20.1%)	30 (37.5%)	198 (18.8%)	<0.001
M	520 (45.9%)	40 (50.0%)	480 (45.6%)	
L	356 (31.4%)	6 (7.5%)	350 (33.3%)	
Remnant stomach	28 (2.5%)	4 (5.0%)	24 (2.3%)	
Stage				
Early	603 (58.6%)	52 (65.0%)	551 (52.4%)	0.029
Advanced	529 (41.4%)	28 (35.0%)	501 (47.5%)	
Depth of invasion				
m	318 (28.1%)	19 (23.7%)	299 (28.4%)	<0.001
sm	285 (25.2%)	33 (41.3%)	252 (24.0%)	
mp	126 (11.1%)	8 (10.0%)	118 (11.2%)	
ss	203 (17.9%)	10 (12.5%)	193 (18.3%)	
sei	200 (17.7%)	10 (12.5%)	190 (18.1%)	
Macroscopic type				
Protruded	192 (17.0%)	11 (13.8%)	181 (17.2%)	0.428
Depressed	940 (83.0%)	69 (86.2%)	871 (82.8%)	
Pathologic type				
Differentiated	610 (53.9%)	37 (46.3%)	573 (54.5%)	0.155
Undifferentiated	522 (46.1%)	43 (53.7%)	479 (45.5%)	
Lymphatic invasion				
Presence	536 (47.3%)	25 (31.3%)	511 (48.6%)	0.003
Absence	596 (52.7%)	55 (68.7%)	541 (51.4%)	
Venous invasion				
Presence	533 (47.1%)	25 (31.3%)	408 (38.8%)	0.182
Absence	599 (52.9%)	55 (68.7%)	644 (61.2%)	
Lymph node metastasis				
Presence	445 (41.6%)	25 (37.3%)	420 (42.1%)	0.169
Absence	622 (58.4%)	44 (63.7%)	578 (57.9%)	
CLS				
Presence	43 (3.8%)	26 (32.5%)	17 (1.6%)	<0.001
Absence	1089 (96.2%)	54 (67.5%)	1035 (98.4%)	
Multiple cancers				
Presence	60 (5.6%)	10 (14.5%)	50 (5.0%)	<0.001
Absence	1007 (94.4%)	59 (85.5%)	948 (95.0%)	

EBV: Epstein-Barr virus; CLS: carcinoma with lymphoid stroma; m: mucosa; sm: submucosa; mp: muscularis propria; ss: subserosa; sei: serosal exposed cancer infiltrating to the adjacent organ.
